# ISIS′ grammar of persuasion of hatred in the article ‘*The Kafir's blood is halal for you, so shed it*’ published in the Rumiyah magazine

**DOI:** 10.1016/j.heliyon.2020.e04448

**Published:** 2020-07-23

**Authors:** Achmad Fanani, Slamet Setiawan, Oikurema Purwati, Maisarah Maisarah

**Affiliations:** a Universitas Negeri Surabaya, Universitas Pesantren Tinggi Darul Ulum Jombang, Indonesia; b Universitas Negeri Surabaya, Indonesia; c Universitas Pesantren Tinggi Darul Ulum Jombang, Indonesia

**Keywords:** Arts and humanities, Linguistics, ISIS, Persuasion, Hatred, Non-muslim

## Abstract

This study is to reveal the types of mood and their speech function realizations in a text (an article) issued in *Rumiyah* magazine entitled ‘The *Kafir's* blood is halal for you, so shed it’. A discourse analysis with a qualitative approach is applied. The results of the analysis are then correlated with Kellermann and Cole's classification of compliance-gaining strategy to see the persuasion strategies applied. The results show that *Rumiyah*, in this text, mainly employs declarative mood to function as statements of opinion, statements of fact, and indirect directives. In terms of Kellermann and Cole's classification, the speech function realizations indicates six strategies of persuasion: The 'nature of situation' to deliver its opinions; the ‘authority appeal’ to present that the opinions and arguments come from very powerful sources; the ‘duty’ to show that hating and killing *mushrikin* (non-Muslims) is an honorable obligation of a Muslim; the 'logical empirical' to clarify and explain Allah's or the Prophet's words or statements; the 'assertion' to state forcefully an obligation or a prohibition of doing something; and the ‘moral appeal’ to get the readers' compliance by appealing to their moral standards. In this text, the statement of fact (TSSS technique) becomes the key element in persuading the readers.

## 1 Introduction

ISIS (Islamic State of Iraq and Syria) is famous for its powerful propaganda activities. Through its media wing, Al-Hayat Media Center, ISIS has been producing various online propaganda publications including *Rumiyah,* an e-magazine, which is written in many languages (e.g., English, French, Bahasa Indonesia) targeting readers outside Arab countries. *Rumiyah* is the replacement of ISIS’ previous e-magazine, *Dabiq.* The e-magazines are used as propaganda means for recruiting foreign recruits from many countries and nationalities (Vergani and Bliuc, 2015).

A study by European Union showed that ISIS, compared with other *jihadist* groups like Al-Qaeda, was the most successful one in recruiting youths from Western countries (Archick et al., 2015). One of the main keys for the success of recruitment is ISIS's powerful language and effective communicative strategies applied in their online propaganda publications such as *Dabiq* and *Rumiyah* (Wignell et al., 2017a, Wignell et al., 2017b).

ISIS’ propaganda involves many narratives and one of them is hatred toward non-Muslims (*mushrikin/kuffar*) as revealed by Azman in her research (2016). But how does ISIS use language to arouse hatred toward non-Muslims? There must be certain linguistic techniques or strategies applied to have the readers comply or agree with them. Wardhaugh (2006, p. 104) stated that a change of topic requires a change in the language used, different linguistic techniques or strategies of persuasion must have been applied for different themes or topics. For instance, persuading a person to hate someone will likely need different linguistic techniques or strategies from persuading the same person to do daily routines as a Muslim.

The main concern of this study was to reveal the types of the mood and speech-function realizations of the clauses used by ISIS to persuade their readers to hate non-Muslims as the enemies of Islam. The text chosen for analysis was the propaganda article entitled ‘The *Kafir's* blood is halal for you, so shed it’ that exists in *Rumiyah* the 1^st^ edition. This is a very provoking article convincing the believers not to be doubtful in killing their enemies (in this article, it refers to *kafir* (a non-Muslim) or *mushrikin* (non-Muslims)). However, the article also explains the kinds of non-Muslims that were allowed to be killed and those who should not be killed (women, children, and *dhimmi kafir* (a person of covenant)). The article tries to convince the readers that the blood of non-Muslims is cheap and has absolutely no price. Therefore, ISIS followers throughout the world are called out to harm and even kill any non-Muslims wherever they meet them. As exemplified in the article, even male flower sellers could be killed because they did not belong to the three groups that should not be killed in war.

### 1.1 ISIS language of propaganda

Azman (2016) revealed three themes that characterize ISIS narratives: (1) ISIS remains strong and lethal; (2) Western non-Muslims should convert to Islam; and (3) ISIS has justifiable reasons for hating their enemies. The three themes were delivered in very powerful language which was mainly used to persuade the readers to agree with them and do an action favored by ISIS.

ISIS′ language has been studied by many researchers around the world, from various viewpoints. However, many researches dealt with how certain words become so influential in ISIS language of propaganda. For example, Vergani and Bliuc (2015) researched the evolution of ISIS’ language in *Dabiq* and found out that: First, affiliation is the important psychological cause for ISIS. Second, the language of ISIS has been using emotions for mobilization. Third, ISIS language relates more with females, to invite not only male fighters but also women. Fourth, the language of ISIS increasingly employs internet jargon (net-speak) to make themselves adjusted to the internet environment and address the youths all over the world.

Another study was conducted by Georges (2015) in which he studied the ISIS linguistic approach to form the notion of *Ummah* (Muslim community) based on the supremacy of Islam. He applied CDA (Critical Discourse Analysis) to analyze the sermon delivered by Abu Bakr Al-Baghdadi on July 4, 2014 in which he announced the formation of the Caliphate (Islamic State) and himself as the Caliph (the leader of the Caliphate). The study revealed that Al Baghdadi incorporated various key Islamic terms into his speech. Furthermore, Georges identified that to make social inclusion and exclusion, Al Baghdadi utilized keywords and phrases like “Muslims,” “mujahidin,” “the Jews,” “your enemy,” and “Ummah of”. Moreover, in Al-Baghdadi's speech, the phrase/words “Islamic State”, “state”, and “khilāfah” were plentifully present because the words/phrases reinforced ISIS's political stratagem through the formation of a Caliphate for rebuilding the Islamic golden age.

However, we cannot only count on power words to influence others. The words should be structured in such a way into appropriate clauses or sentences to make them more powerful. In other words, grammar is crucial in persuasion (Power, 1998; Hogan and Speakman, 2006). In ordering, directing, guiding, or persuading others successfully, one should have right choices of clause/sentence patterns. Henceforth, this current study would deal with the exploration of clause/sentence patterns utilized by ISIS in their propaganda magazine ‘*Rumiyah’*.

Because the grammatical analyses of ISIS′ language are rarely conducted and the analysis of mood type in political propaganda/persuasive texts mostly focuses on discovering the types of mood in persuasive texts, this present study would like to address the research gap that is investigating the grammar (mood and speech-function realizations) of ISIS’ persuasion in one of its article. The result of the analysis would complement the results of previous studies on ISIS language which mainly dealt with the analysis of power words or text themes.

### 1.2 Mood system in systemic functional linguistics

Hallidayan Systemic Functional Linguistics (SFL) is suitable for evaluating the language of persuasion/propaganda since one of its concerns is the interpersonal meaning (mood) analysis. SFL stresses that the relationship between speakers/authors and their audience/readers has an effect on the language the speakers use. In SFL, the usage of language to relate with others and their usage to negotiate interactions and to convey opinions and attitudes is examined at the level of clause (Halliday and Matthiessen, 2004). The clause can be a functional proposition which is used to inform or question or a proposal which is used to give orders or make offers. Besides, a clause articulates one's judgment and attitude towards whoever he/she is handling and what he/she is talking about (Halliday, 2014; Eggins, 2004).

The mood system is a clause structure that reflects certain types: indicative or imperative. The indicative mood reflects that something is or is not the case; whereas the imperative mood reflects the author's/speaker's desires that something should happen. The system is closely correlated with the language of persuasion because the mood of a clause expresses the author's/speaker's attitude about the state of being of what the sentence illustrates (Halliday in Fanani et al. (2019)). The author's/speaker's attitude of something will certainly affect the effectiveness of persuasion they work on.

The indicative mood has two types: declarative and interrogative. The typical speech-function realization of a declarative is as a statement (facts, opinion, etc.) that serve to provide information; while interrogatives typically functions to request information (Halliday, 2014; Eggins, 2004). Imperative Mood is the mood of the verb and "the principal mood of will and desire" (Lyons, 1977). Imperatives have the typical speech-function realization as orders, requests, and directives (Eggins, 2004). The imperative mood does not occur in subordinate clauses or subordinate questions because basically, this kind of mood is performative (Palmer, 2001). However, the mood type is not always one on one with its typical speech functions. A declarative or a question, for example, may have a directive function as in ‘You should go now’ or ‘Can you go now?’

### 1.3 Persuasion: definition

A speaker or an author usually utilizes some linguistic devices in delivering his speech or in writing in order to persuade the audience or the readers. In persuasion, verbal or written words and visual devices play significant roles in altering the targets’ mind, attitude or behavior.

There are different definitions of persuasion. Gass and Seiter (2010) define persuasion as an effort to influence a person's beliefs, attitudes, intentions, motivations, or behaviors. Simons et al. (2001) give a more general definition of persuasion that is human communication designed to influence the autonomous judgments and actions of others. Dillard and Pfau (2002) proposes a more complicated definition of persuasion that is a symbolic transaction, which uses reason and/or emotional appeals to alter behavior. Although the definitions are delivered in different words, they share one thing in common that is changing the behavior and thoughts of others. Hence, in this current research, persuasion is defined as any linguistic devices structurally employed by the author for changing the readers' actions (behaviors), attitude, or thought.

### 1.4 Persuasion: types of persuasive texts

A persuasive text is also understood as an argumentative or expository text. In this research, this kind of text is defined as a form of rhetorical production involving the identification of a thesis or claim, the establishing of supportive evidence, and the evaluation of warrants that connect the thesis, evidence and subject matter of the argument (Newell et al., 2011).

Coffin (2004) distinguishes two terms in persuasive texts: analytical and hortatory expositions. An ‘analytical exposition’ text is aimed at “persuading that” whose social function is to convince the readers or audience that something is imperative and needs to get attention. Besides, it also functions to persuade the readers that the opinion is true and backed up by robust arguments. A ‘hortatory exposition’ is aimed at “persuading to” which convinces the readers to do something in a certain way – to perform a social action. In this kind of text, the author provides his opinions and arguments to support the topic of the text. The recommendation is commonly put at the end of the text.

### 1.5 Persuasion: Kellermann and Cole's 64 compliance-gaining strategies

Compliance gaining is a part of persuasion. It takes place when a person purposely convinces the other to do or get something that he/she might have not done or get otherwise. Kellermann and Cole (1994) formulate 64 compliance gaining strategies as an effort to categorize more than 820 previous strategies. The strategies are described in the following table (see Table 1).

**Table 1:** Kellermann and Cole's 64 compliance gaining strategies.

No	Strategy	Example	No	Strategy	Example
1	Actor Takes Responsibility	Is there anything I can do with this? May be I can help.	33	Esteem (Positive) by Actor	If you go now, I'll be so thankful to you.
2	Altercasting (Negative)	Only a selfish person would not donate to the program.	34	Expertise (Negative)	If you do this, you will be very disappointed.
3	Altercasting (Positive)	Great men like you would never miss the opportunity.	35	Expertise (Positive)	When you go to Indonesia, be sure to visit Bali. It is a spectacular island.
4	Altruism	May I ask your help with this? I am really confused with this.	36	Hinting	I'm bored. [indicating 'let's go home now']
5	Assertion	I want that work finished this night.	37	I Want	I want to go home now.
6	Audience-Use	A child asks his mother to give him a candy when there are other people there. His mother feels powerless to say no to give him one.	38	Invoke Norm	Donate charitably, like the others do!
7	Authority Appeal	It is your mother's order, obey it.	39	It's Up to You	I'd really like to finish it now, but it's up to you.
8	Aversive Stimulation	Go now! Get it now! Don't wait, just do it now!	40	Logical Empirical	You must be realistic to take the decision. Let's look at the data.
9	Bargaining	If you want me to still be your friend, you'll do this.	41	Moral Appeal	Don't shop there. The new shop sells many illegal products.
10	Benefit (Other)	I don't want it for myself -- it's for my child.	42	My Concern for You	I am really worried about you. How if I take you to your doctor now?
11	Benefit (Self)	If you help me with this, I'll get a better score on Math.	43	Nature of Situation	Excuse me, I know that you love one another, but kissing in public like that is inappropriate here.
12	Benefit (Target)	If you study hard now, you will be able to go to a reputable college.	44	Negative Affect	Are you stupid? Watch me. Just do it like this!
13	Challenge	The customer looks unhappy. I know you are great in dealing with unhappy people.	45	Not Seek Compliance	'Never ask! Just do it, okay.'
14	Compliment	You always have good ideas. Could you help me find the answer for this puzzle?	46	Persistence	Hello again, Mr. Smith. I phone you again to know whether you have already got time to discuss my proposal.
15	Compromise	A sales person agrees to give an extra discount for an order placed today.	47	Personal Expertise	I'm very good at math. I think I can help you with this math problem.
16	Cooperation	I'm having trouble with your late response on this problem. Why don't we cooperate and work something out?	48	Positive Affect	Congrats for your achievement. And of course it also means that we will have a big party.
17	Criticize	You have taken a stupid way. You must do it again.	49	Pre-Giving	I give her a beautiful gift before I ask her to marry me.
18	Debasement	I really don't know anything about this place. Can you help, just a little?	50	Promise	I promise you to well behave. Can you buy me the toy now?
19	Debt	I've contributed much for this institution. I think I deserve a promotion.	51	Promote Task	Can you fill out the form now so we can leave on time?
20	Deceit	A sales person misled the new customer into believing that the car was worth more than it really was.	52	Self-Feeling (Negative)	Doing it right now is very beneficial. You'll feel very sorry if you miss it.
21	Direct Request	Can you do this for me?	53	Self-Feeling (Positive)	This is your last chance. If you take it, you'll feel very pleased.
22	Disclaimer (Norms/Rules)	Oh come on. Let's just submit the paper. Don't worry about the review requirement.	54	Suggest	It'd be better that you just ask her out. Just say 'Would you like to go out with me tonight?'
23	Disclaimer (Other)	You are the only one who is unselfish. You have to help him.	55	Surveillance	You can't waste your money like this. I'm going to have a closer eye on your spending.
24	Disclaimer (Self)	Actually, I really don't know want to bother you, but the situation makes me ask your help.	56	Third Party	A student asked his teacher to have one of his friends forgive him.
25	Disclaimer (Target)	I know you may don't have time to do this, but I have to ask you to do so.	57	This Is the Way Things Are	You ask me why you must do that? Ok, I tell you. It's the rule, my friend.
26	Disclaimer (Task)	Don't worry, it's not painful. It will only take a few seconds.	58	Thought Manipulation	You said I could submit the proposal today! You said so.
27	Disclaimer (Time)	You can finish the task later. I want you to help me with this now.	59	Threat	If you don't finish it on time! I won't give you a break.
28	Duty	The manager said, “I want it now. You must get me one.”	60	Value Appeal	Being truthful is important. Well, can you tell me the truth about it?
29	Equity	It's now Jack's turn. You have had it before.	61	Warning	Mind your step! It's a bit slippery there.
30	Esteem (Negative) by Others	If you don't do this, your friends will think that you are a liar.	62	Welfare (Others)	If you don't show up now you'll ruin the occasion.
31	Esteem (Positive) by Others	If you get it, your wife will be very thankful to you.	63	Why Not?	Let's do it. Why not?
32	Esteem (Negative) by Actor	I think it is not a good decision. If I were you, I wouldn't do that.	64	Your Concern for Me	If you really want to help me, you'll lend me some money.

## 2 Method

**Figure 1:**
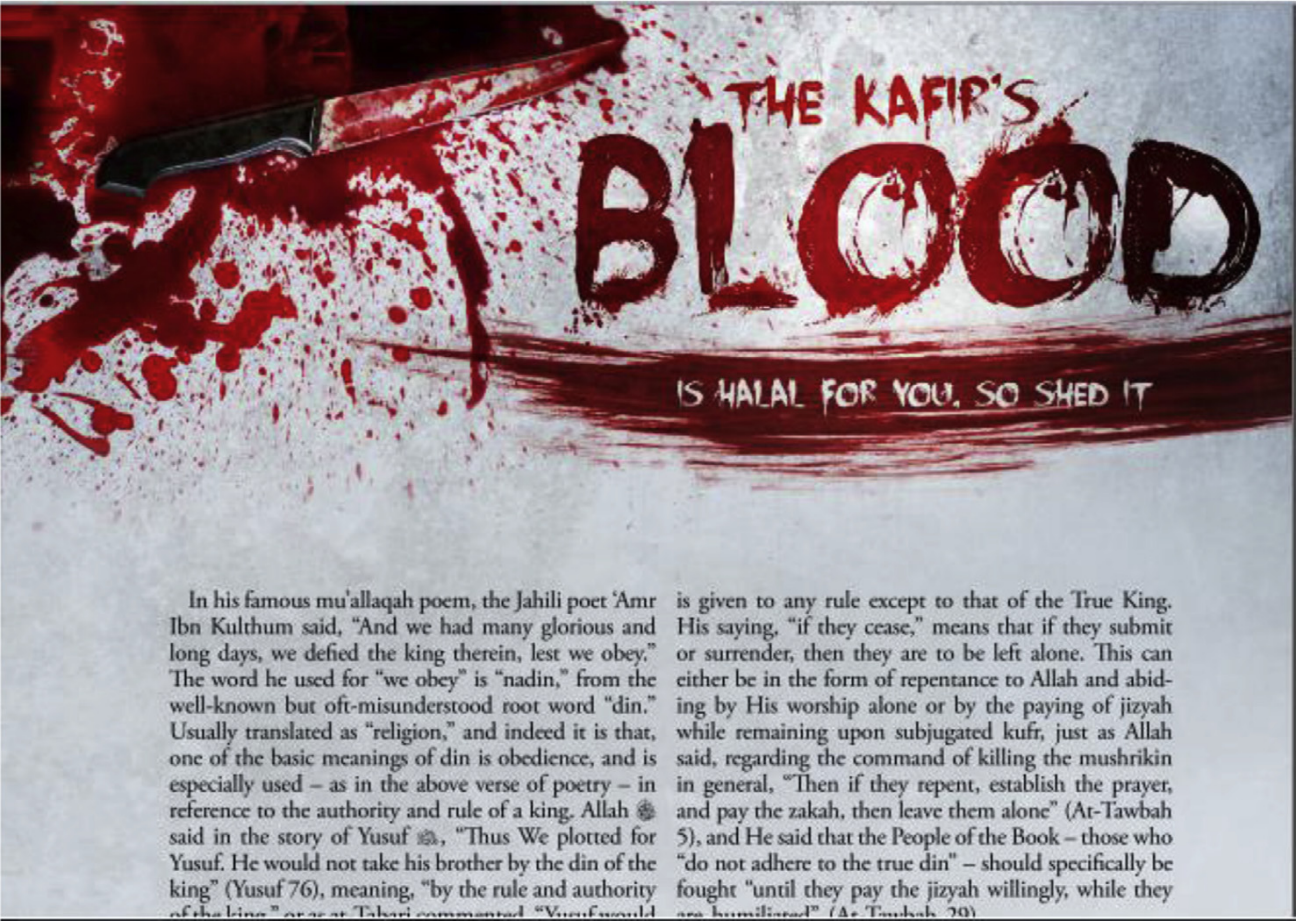
The first page of the analyzed article (*Rumiyah* the 1^st^ edition, Dhul-Hijjah 1437).

This current research applied a discourse analysis which was qualitative in nature to see how the grammar was deployed for the purpose of persuading the readers to hate *mushrikins*. The data source (the article ‘The Kafir's Blood Is Halal for You, So Shed It’) was taken from *Rumiyah* magazine the 1^st^ edition, Dhul-Hijjah 1437 (Islamic year) (See Figure 1). In the article, there were 64 major clauses to analyze. In this research, each simple sentence or complex sentence was counted as one clause. One compound sentence consisting of two major clauses was calculated as 2 clauses, depending on the number of main clauses that construct the sentence. The data (major clauses) were then analyzed to see the types of the mood and the speech-function realizations of the clauses to build the persuasive clauses. The discussion was emphasized on how the key findings of SFL patterns (mood types and speech function realizations) reflect the persuasive strategies applied in the text.

## 3 Results and discussion

### 3.1 Results

Table 2 shows the mood types and their speech function realizations of the main clauses that are present in the text. From the table, it can be seen that there are 64 main clauses written in declarative mood (63 clauses) and interrogative mood (1 clause).

**Table 2:** The mood type of clauses and speech function realization in the text.

Mood type	Speech Function Realization	Technique∗	Examples
Declarative	Statement (opinion)	• PMS	• Usually translated as “religion,” and indeed it is that, one of the basic meanings of din is obedience, and is especially used – as in the above verse of poetry – in reference to the authority and rule of a king (Clause 3)
		• GEOS	• This is because they are from the same people who are at war [with the Muslims] (Clause 55) • Thus, anyone who is neither a Muslim nor a dhimmi kafir (while still a tyrant against himself, deserving both hatred and humiliation) is a hostile tyrant deserving aggression (Clause 12) • So the duty to fight the tyrants – the mushrikin – is clear and established (Clause 15)
	Statement (fact)	• TSSS	• Ash-Shafi'i said, “And the kafir's blood is not spared until he becomes a Muslim” (Al-Umm) (Clause 38) • And regarding the dhimmi … the Prophet said, “Whoever kills a person of covenant shall not smell the fragrance of Jannah, which can be found for a distance of forty years” … (Clause 24)
	Indirect directive	• PODS	• One of these great principles is that all people must be fought until they accept Islam or come under a shar'i covenant (Clause 21) • So the priest and wandering ascetic who mix with the people are to be killed, … (clause 54)
		• PPDS	• This principle establishes the prohibition of shedding Muslim and covenant-bound kafir blood as well as the permissibility of shedding the blood of all other *kuffar* (Clause 22) • But it is not permissible to kill anyone who is not from the people who are at war … (Clause 53)
		• GS	• Lest someone think this is a strange, new opinion, it should be known that this is the stance of the Sahabah and the greatest scholars of the Ummah (Clause 33)
Interrogative	Indirect directive	• asking	• How can the disbelievers ever dream of safety and security while Muslims suffer anywhere in the world and while the rule of Allah is mockingly replaced by manmade monstrosities of democracy? (Clause 64)

∗ PMS (presenting the meaning/the characteristic of something); GEOS (giving an evaluative opinion of something); TSSS (telling about someone saying something); PODS (presenting the obligation of doing something); PPDS (Presenting prohibition of doing something); GS (Giving suggestion).

The declarative mood in this text isused to exchange commodities (information) to the readers. In this context, they are used to make statements with three different functions: statements of opinion (32 clauses), statements of fact (23 clauses), and indirect directives (8 clauses);

In composing statements of opinion, there are two techniques applied:

(1) Presenting the meaning/the characteristic of something (PMS)
(2) Giving an evaluative opinion of something (GEOS)

The first technique is used to present the meaning/definition/characteristic of something (PMS) as in Clause 3 where *Rumiyah* defines the word ‘din’. It is defined that ‘*din’* means obedience to the authority and rule of a king. Out of five clauses that use the technique, one clause (Clause 03) contains the main information of the paragraph, which means it contains the main idea in the corresponding paragraph. In this instance, it asserts the obedience to authority as the meaning of *din.* As for the rest of the clauses, they function as supporting information to the main ideas of the corresponding paragraphs.

In the second technique, giving an evaluative opinion of something (GEOS), some clauses, phrases or adjectives indicating an evaluation of certain things are utilized. For example, in Clause 55 the complement of the clause (‘because they are from the same people …’) represents an evaluative opinion about something mentioned earlier. In this instance, it is the reason why the priest and wandering ascetic have to be killed. Besides, a categorization or classification to present an evaluative opinion is made as in Clause 12. In this instance, *Rumiyah* sends its opinion by categorizing anyone who is neither a Muslim nor a *dhimmi kafir* (a person of covenant) as bad people (hostile tyrants) who, therefore, deserved aggression.

Out of 27 GEOS clauses, six clauses contain the main information of the corresponding paragraphs while the other 21 clauses function as supporting information. The clauses with the main information assert the main idea of persuasion (the main content of discussion). For example, Clause 15 asserts to the readers the main idea that fighting the *mushrikin* (non-Muslims) is the duty of a Muslim while clauses 16 to 18 in the corresponding paragraph give supporting information about the idea contained in clause 15.

In composing the statements of fact, *Rumiyah* only applies one technique that is ‘telling about someone saying something’ (TSSS). The technique is mainly used to support *Rumiyah's* argumentation presented beforehand. For example, Clause 38 supports *Rumiyah's* opinion that shedding the blood of *mushrikin* is permissible. In this technique, *Rumiyah* copiously applies direct quotations.

Out of 16 TSSS clauses, one clause contains the main information while the other 15 clauses contain supporting information. The clause with main information asserts the main idea of persuasion (the main content of discussion). For example, Clause 24 asserts to the readers the main idea of the corresponding paragraph that killing the *dhimmi* (a person of covenant) is prohibited while Clause 25 in the corresponding paragraph gives supporting information about the idea contained in Clause 24.

In some instances, *Rumiyah* uses a declarative mood to function as an indirect directive, indirectly asking the readers to do something. To do this, three techniques are applied:

(1) Presenting the obligation of doing something (PODS)
(2) Presenting the prohibition of doing something (PPDS)
(3) Giving suggestion (GS)

Firstly, the indirect directive is formed by utilizing the modal verb ‘must’ and ‘to be+base verb’ construction indicating an obligation (PODS). For example, in Clause 21, *Rumiyah* asks the readers indirectly to do something, as indicated by the complement of the clause (*that all people must be fought…*). In this instance, it asks them to fight all people until they accept Islam or come under a *shar'i* (Islamic law) covenant. The clause is comparable to a more blatant imperative “Fight all people until….”

*Rumiyah* also applies “to be + base verb” construction indicating an obligation. For example, in Clause 54, it presents the obligation of the Muslims to do something, as indicated by the presence of ‘to be + base verb’ construction (to be killed). In this example, killing the priest and wandering ascetic who mix with the people is a must to do.

Out of five PODS clauses in the text, three clauses function as the main information of the paragraphs while two clauses function as supporting information. As they contain the main information, they assert the main idea of persuasion (the main content of discussion). For example, clause 21 asserts to the readers the main idea that fighting all people until they accept Islam is the obligation of a Muslim. The idea in the clause is supported by clause 19, 20, 22, and 23 in the corresponding paragraph.

Secondly, the indirect directive is formed by presenting a prohibition of doing something (PPDS)*.* In some declarative sentences, *Rumiyah* disallows the readers from doing something. This kind of prohibition is equivalent to a negative imperative (e.g., Don't do it). For example, in Clause 22 *Rumiyah* asks the readers not to do something (i.e., shedding Muslim's and covenant-bound *kafir's* blood). The clause is equivalent to a more forceful negative imperative “Don't shed the blood of Muslim and covenant-bound *kafir*).

Out of two PPDS clauses in the text, one clause contains the main information of the corresponding paragraphs while another clause functions as supporting information. The clause with main information asserts the main idea of persuasion (the main content of discussion). For example, Clause 53 asserts to the readers the main idea that killing anyone who is not from the people who are at war is not permissible. The idea is supported by clauses no. 54 and 55 in the corresponding paragraph.

Thirdly, the indirect directive is formed by suggesting something (GS) as indicated by the modal verb ‘should’. In *C*lause 33 *Rumiyah* suggests the readers to understand that hating and killing non-Muslims is the stance of the *Sahabah* and the greatest scholars of the *Ummah*. The clause contains the main ide of the paragraph and is supported by clauses no. 34 to 41.

Besides declarative mood, another mood is also present in this text. One clause can be categorized as an interrogative mood which functions as an indirect command. In *C*lause 64, *Rumiyah* uses a modalized interrogative (H-Question with can). The clause is a reflective question and is used to close the text. Though it is superficially a question, it actually indicates a request to do something (i.e., to make the disbelievers feel unrest). The clause is comparable to a simpler construction of “How can *someone* do something, while *someone else* is in certain situation? When a speaker asks a hearer, “How can they buy your product, while the price is too high?” it implies that he asks the hearer to reduce the price.

## 3.2 Discussion

There are two interesting SFL aspects to discuss in the results above. *Firstly*, the text is dominated by the declarative mood; and *secondly*, the mood is divided into three different speech functions: statement of opinion, statement of fact, and indirect directive. These different speech functions possess special roles in the persuasion of hatred.

The dominance of the declarative mood in the text shows that *Rumiyah* tends to position itself as a carrier of information to the readers, i.e., giving statements. With this mood, it can shorten the distance with its readers; hence can directly send the information. This is certainly different from interrogative or imperative moods that require responses from the readers or audiences to see the effectiveness of a proposition (Halliday and Matthiessen, 2004).

However, in line with Ayoola's research (2013) which indicated that the interpersonal meaning of a structure was not always consistent with its lexicogrammar, so are the realizations of the speech functions of the declarative mood in this text. They do not always correspond to the typical speech function of a declarative mood, namely as a statement. Some declarative mood functions as indirect directives expressing a command or prohibition of doing something. For example, in “One of these great principles is that all people must be fought until they accept Islam or come under a *shar'i* covenant (Clause 21)”, *Rumiyah* asks every Muslim (including the readers) to fight all people until they accept Islam. Although it is delivered in a declarative mood, essentially it is an order to do something and is comparable to a more robust command ‘Fight all people until they accept Islam.” Another example is in Clause 53 (“It is not permissible to kill anyone who is not from the people who are at war ….”). In this instance, *Rumiyah* prohibits the readers to do something as indicated by the expression “It is not permissible to kill anyone”.

Within the text, the declarative mood also functions as a statement of opinion. In this case, they contain emotive words (e.g., hostile, tyrant, impure, be slain, permissible, impure) which are attached to the subject, predicator, complement, or adjunctive elements. This is in line with Vergani & Bliuc's research (2015) which revealed that ISIS′ language had been using emotions for mobilization. According to Martin and White (2005) the negative-sensed words reflect negative assessment (appreciation) to others. In this text, such negative assessment is aimed at arousing hatred toward non-Muslims; hence gives permissibility of doing something bad to them. For example, the phrase ‘entirely impure’ gives negative quality to non-Muslims (*mushrikin*). In “*Indeed, ‘Umar was correct, as the mushrikin are entirely impure, as Allah said, “….”* (Clause 35), *Rumiyah* arouses the readers’ disgust to non-Muslims because they are dirty completely. Similarly, the abundant use of the adjective ‘permissible’ or ‘*halal*’ in the text is commonly used to provide a positive judgment of the blood-shedding of non-Muslims. By presenting such appreciation and judgment, the readers are convinced that the blood of non-Muslims (*mushrikin*) is dirty, as what Allah said, hence it is permissible to shed.

In Kellermann & Cole's classification, giving a negative evaluation to non-Muslims can be classified as the 'nature of situation' strategy since *Rumiyah* positions itself as the one who well understands about the matter of discussion, hence the nature of something. As an expert, *Rumiyah* gives definitions or meaning to something, or even gives interpretation to Allah's and the Prophet's statements based on factual facts. As an expert, any definition or evaluation it makes will sound valid or accurate since they are based on robust facts (Clark et al., 2011).

Within this text, *Rumiyah* also plentifully quotes the statements from Allah, the Prophet, or Muslim scholars. This is in agreement with the results of Georges's research (2015) which revealed that the statements from Allah and His prophet are commonly used in the ISIS′ narrative of propaganda. The statements containing words indicating orders and prohibitions (e.g., command of Allah, permissible, prohibition) are actually instructions to the readers which are delivered indirectly. In Martin & White's appraisal theory (2005), this technique is called blurring of semantic boundaries. In this way, *Rumiyah* reduces its personal investment in commanding the readers, avoiding subjective instructions and resorts to using words that indicate instruction or prohibition.

In Kellermann & Cole's classification (1994), quoting statements from God and the prophet is a form of 'authority appeal' strategy that has powerful effect because people have a tendency to obey the ideas or instructions of authorities; people trust what they say. Fessenden (2018) clarifies that the authority principle is an example of the human preference to use judgment heuristics, which suggests that those in positions of authority may have better knowledge and power, and therefore, acting in accordance with them will bring about a positive result. Through the authority appeal strategy, it is explained that spilling the blood of unbelievers or hating them is a command from the very powerful authority (i.e., Allah and the Prophet) which, therefore, must be fulfilled. Further, Mulholland (1994) says that reputable authorities would help an influencer in doing his/her job which implies that any disapproval to them should cease. To be powerful, the authority must be the prominent and worth hearing ones. For instance, one can cite Allah or Prophet Muhammad to persuade Muslims, or cite Chomsky to persuade linguists.

Besides, this strategy also denotes the implementation of another compliance-gaining strategy that is 'duty', which is used to show that hating and killing non-Muslims is an honorable obligation of a Muslim that must be done earnestly. In Straker's (2002) opinion, much of our lives are guided by laws and rules, from national laws and rules to religious laws, company rules, social norms, personal standards, and family ways. Fulfilling any of these laws, rules or regulations is a duty, which must be fulfilled. Defying a duty would bring about bad outcomes.

Quoting the Muslim scholars' statements and explanations is an implementation of 'logical empirical' strategy. It is utilized to clarify the statements from Allah or Prophet Muhammad. Clark (2008) explains that direct quotation may intensify opinions by directing the readers to a recognized authority. By this strategy, *Rumiyah* quotes elucidations from Muslim scholars to make the presented argumentation – shedding the blood of non-Muslims – have a robust scientific foundation. The cited Muslim scholars are commonly well-known persons in Islam such as Ibn Hazm, Ash-Shafi'i, etc. whose opinions and explanations are usually observed. For example, in Clause 45 the statement from an expert (Ibn Hazm) is used to provide a scientific basis about the acceptability of shedding the blood of non-Muslims.

The declarative moods that serve as indirect directives indicate the obligations and prohibitions of doing something. This is a kind of 'assertion' strategy where an obligation or a prohibition of doing something is straightforwardly asserted. The use of many words indicating an obligation (e.g., must, be to, command) implies that *Rumiyah* would like to strengthen or intensify the meaning of a clause. In Martin & White's appraisal theory (2005), it is an intensified judgment of force. For instance, Clause 08 directly states the obligation of a Muslim to fight the non-Muslims because it is Allah's command. The phrase ‘the command of Allah’ and the construction of ‘is to’ in this clause indicates an assertion of obligation.

An assertion is a stylistic method or strategy containing a forceful statement, a robust or assertive, and positive declaration about a belief or a fact. An assertion is frequently an indirect appeal to authority in that it makes the supposition that the one making the assertion is a knowledgeable person or has a position of unquestionable recognized authority (Straker, 2002). Assertion with the directive function will make the readers respond and act more immediately and observe more devotedly, particularly when the command comes from a very authoritative source (e.g., Allah, Prophet).

Within this text, a 'moral appeal' strategy is also used to gain the readers' compliance. Straker (2002) states that people regularly use their moral criterion as an essential measure of recognizing their personality, betraying their morals is basically a form of identity destruction, decreasing their very being. In other words, people frequently employ a moral standard to become virtuous people. Essentially, with this strategy, *Rumiyah* gets the readers' compliance by appealing to their moral standards and convinces them that instigating harm to non-Muslims is the fair and right thing to do. This strategy awakens the readers’ empathy toward the disgraceful condition of the other Muslims everywhere in the world and toward the law and rule of Allah which is mocked and scorned by the enemies of Islam (non-Muslims).

### 3.3 Clause composition and persuasion

In this section, how *Rumiyah* composes the paragraphs is illustrated. It is described how the clauses relate one another to form a paragraph and how they work in persuasion. The techniques used in composing the clauses indicate how the clauses are deployed to persuade the readers.

This text is not purely analytical (persuading that) or hortatory (persuading to) rather a combination between the two. It means that on one side, it is aimed at persuading the readers that non-Muslims deserve to hate (analytical), while on the other side, it is also aimed at persuading the readers to kill certain kind of non-Muslims (hortatory)*.* The clauses in the text well represent the purpose. The following table illustrates how the clauses relate one another in each paragraph in order to persuade the readers.

Table 3 illustrates that there are eight main ideas discussed in the text; Definition of Din, Introductory to fight *mushrikin* (non-Muslims) as the obedience to Allah's rule, Exception of fighting a certain kind of *kafir* (a non-Muslim)*,* Duty to fight the tyrants, obligation of shedding the blood of a *kafir,* Prohibition of killing the *dhimmi* (a person of covenant)*,* Exception of killing women and children, and permissibility of shedding the blood of a *kafir.* Out of them, the underlined idea of this text is strengthening the obligation/permissibility of shedding the blood of *mushrikin,* which is discussed in the most parts of the text.

**Table 3:** Clause composition in each paragraph.

Clauses	Main idea	Technique (clause)
Clause 1 – Clause 5	Definition of Din	TSSS (1,4,5), PMS (2,3)
Clause 6 – Clause 8	Introductory to fight *mushrikin* as the obedience to Allah's rule	PODS (6,8), TSSS (7)
Clause 9 – Clause 14	Exception of fighting a certain kind of *kafir*	PMS (9,10)*,* GEOS (11,14)
Clause 15 – Clause 18	Duty to fight the tyrants	GEOS (15,16,17)*,* TSSS (18)
Clause 19 – Clause 23	Strengthening the obligation of shedding the blood of a *kafir*	GEOS (19)*,* PMS (20)*,* PODS (21), PPDS (22)*,* TSSS (23)
Clause 24 – Clause 25	Prohibition of killing the *dhimmi*	TSSS (24), GEOS (25)
Clause 26 – Clause 32	Strengthening the obligation of shedding the blood of a *kafir*	GEOS (26,27,29,30,31), TSSS (28,32)
Clause 33 – Clause 41	Strengthening the obligation of shedding the blood of a *kafir*	GS (33), GEOS (34,35,36,40)*,* TSSS (37,38,39)
Clause 42 – Clause 43	Exception of killing women and children	TSSS (42), GEOS (43)
Clause 44 – Clause 48	Strengthening the obligation of shedding the blood of a *kafir*	TSSS (44,45,46,47,48)
Clause 49 – Clause 52	Strengthening the permissibility of shedding the blood of a *kafir*	GEOS (49), TSSS (50,51,52)
Clause 53 – Clause 55	Strengthening permissibility of shedding the blood of a *kafir*	PPDS (53)*,* PODS (54), GEOS (55)
Clause 56 – Clause 57	Strengthening the permissibility of shedding the blood of a *kafir*	GEOS (56)*,* TSSS (57)
Clause 58 – Clause 63	Strengthening the permissibility of shedding the blood of a *kafir*	PODS (58), GEOS (59,60,61,62,63)
Clause 64	Closing	Asking (64)

In this section, the clauses that make up each idea represent a distinctive pattern for persuading the readers. Most of the paragraphs are composed of several clauses that function differently. The general pattern is that any statements of opinion or indirect directive are accompanied by a statement(s) of fact with the technique of telling about someone saying something (TSSS). This indicates that such clause has significant role in persuading the readers. Exception is the relations of clauses 9–14 and 53–55. In the clauses of 53–55 *Rumiyah* does not use any statement of fact in them. However, in the clauses of 9–14, *Rumiyah* does not apply TSSS technique directly, but in clause 10 it indirectly cites Allah's statement and puts it in the adjunctive element, “*…., just as Allah said, regarding the command of killing the mushrikin in general, …, and He said that the People of the Book – those who “do not adhere to the true din” – should specifically be fought “until they pay the jizyah willingly, while they are humiliated” (At-Tawbah 29)* (Clause 10).

The technique of TSSS provides a strong basis of argumentation for the previous opinions or instructions represented by other clauses. For example, in defining the word ‘din’, *Rumiyah* does not only use its own statement (Clause 3) but also bases it on the other's opinion (i.e., at-Tabari) as in Clause 4 and 5. The clauses, more specifically Clause 5, strengthen *Rumiyah's* opinion that the basic meaning of din is obedience to authority.

Similarly, Clause 6 to Clause 8 introduce one of Allah's rules that must be fulfilled as a manifestation of obedience to Allah – the King of mankind, the King of the Day of Recompense, the True King. *Rumiyah* mentions the religious obligation to fight non-Muslims by quoting Allah's statement “*and He said, “And fight them until there is no fitnah and the din is for Allah. But if they cease, then there is no aggression except against the tyrants”* (Clause 7). Because the word ‘din’ has been defined as the obedience to authority, the clause presents what obligation to fulfill. In this instance, the readers are convinced to fight the *mushrikin* because Allah, the King of mankind, says so. Again, the technique of telling about someone saying something becomes the key in persuading the readers. The direct quotation technique brings about an impression that Allah does say so.

Another example is when *Rumiyah* presents the obligation of shedding the blood of a *kafir* – as the key message of this text – e.g., in Clause 19 to Clause 23, *Rumiyah* uses a direct statement from prophet Muhammad to support its opinions “*The Prophet said, “I have been ordered to fight mankind until they say that there is no god except Allah and that I am the Messenger of Allah, ….*” (Clause 23). In the previous clauses (19–22), *Rumiyah* gives opinions about the obligation/permissibility to shed the blood of a *kafir*. The Prophet's statement then strengthens its opinion and makes it more powerful argumentation.

The TSSS technique provides a solid basis for the presented arguments because all of the parties quoted are the strong and powerful authorities in Islam (Allah, Prophet Muhammad, or respected Muslim scholars). Therefore, it is almost impossible for a devout Muslim to oppose Allah's or His prophet's commands or explanation. In persuasion strategies, this method is called ‘authority appeal’ (Kellermann and Cole, 1994). Everyone tends to comply with whatever is said by those they respect and have power over them. A good student will tend to obey his teacher's orders; a servant will obey his master's orders, and so forth. This is why the opinion or any order to hate or kill non-Muslims will likely to be obeyed or accepted by ‘devout’ Muslims without any reserve because challenging such opinions or instructions means challenging Allah and His Messenger, which is, of course, unacceptable and sinful.

In short, clauses that function as statements of opinion or indirect directives contain *Rumiyah's* personal ideas. Each opinion or command (indirect) is always based on a strong basis of argumentation derived from powerful authorities (Allah, Prophet Muhammad, respectable Muslim scholars). In other words, opinion clauses and indirect directives are always intertwined with the statements of fact with TSSS technique.

## 4 Conclusion

Based on the findings and discussion above, it can be concluded that in this text the declarative mood is mainly used to convince the readers that *mushrikin* (non-Muslims) deserve to be hated and killed (analytical exposition) and that the readers (Muslims) should hate and kill them (hortatory exposition). The dominance of the declarative mood in the text shows that *Rumiyah* tends to position itself as a carrier of information to the reader, i.e., giving statements. With this mood, *Rumiyah* can shorten the distance with its readers; hence can directly send the information. The declarative moods are mainly utilized to present opinions, facts, and indirect instructions. In delivering opinions, many emotive words are utilized to evoke the readers’ negative emotions to non-Muslims. The facts are mainly delivered by citing the words or statements from Allah, the Prophet, or Muslim scholars through a direct quotation technique. The indirect directives (instructions) are presented by presenting obligation and prohibition of doing something through the declarative moods.

In terms of Kellermann and Cole's classification, *Rumiyah* applies six strategies in this text: The 'nature of situation' strategy to deliver its opinions; the ‘authority appeal’ strategy to present that the opinions and arguments come from very powerful sources; the ‘duty’ strategy to show that hating and killing non-Muslims is a honorable obligation of a Muslim; the 'logical empirical' strategy to clarify and explain Allah's or the Prophet's words or statements; the 'assertion' strategy to state forcefully an obligation or a prohibition of doing something; and the ‘moral appeal’ strategy to get the readers' compliance by appealing to their moral standards. In this text, the statement of fact (direct quotation) becomes the key element in persuading the readers.

## Declarations

### Author contribution statement

A. Fanani: Conceived and designed the analysis; Analyzed and interpreted the data; Wrote the paper.

S. Setiawan: Conceived and designed the analysis; Performed the experiments.

O. Purwati: Analyzed and interpreted the data.

M. Maisarah: Contributed reagents, materials, analysis tools or data.

### Funding statement

This work was supported by 10.13039/501100014538LPDP (Indonesia Endowment Fund for Education) , Ministry of Finance, Republic Indonesia [grant number 20161141080700].

### Competing interest statement

The authors declare no conflict of interest.

### Additional information

No additional information is available for this paper.

## Figures and Tables

**Table:** 

Data no.	Clause	Mood type	Speech function realization	Technique	Strategy of persuasion reflected	Function in Parapgraph
Clause 01	In his famous mu'allaqah poem, the Jahili poet ‘Amr Ibn Kulthum said, “And we had many glorious and long days, we defied the king therein, lest we obey.”	Declarative	Statement of fact	telling about someone saying something	Logical-empirical	Supporting information
Clause 02	The word he used for “we obey” is “nadin,” from the well-known but oft-misunderstood root word “din.”	Declarative	Statement of opinion	presenting the meaning/the characteristic of something	Logical-empirical	Supporting information
Clause 03	Usually translated as “religion,” and indeed it is that, one of the basic meanings of din is obedience, and is especially used – as in the above verse of poetry – in reference to the authority and rule of a king.	Declarative	Statement of opinion	presenting the meaning/the characteristic of something	Nature of situation	Main information
Clause 04	Allah said in the story of Yusuf, “Thus We plotted for Yusuf. He would not take his brother by the din of the king” (Yusuf 76), meaning, “by the rule and authority of the king,” or as at-Tabari commented, “Yusuf would not take his brother by the rule, judgment, or obedience of the king of Egypt.”	Declarative	Statement of fact	telling about someone saying something	Logical-empirical	Supporting information
Clause 05	He then mentioned the various statements of the Salaf suggesting that “din” in this ayah means sultan (authority), qada (judgment), and hukm (rule), ending his commentary by saying, “And the basis of din is obedience.”	Declarative	Statement of fact	telling about someone saying something	Logical-empirical	Supporting information
Clause 06	As for our King – the King of mankind, the King of the Day of Recompense, the True King – then we have entered into His din and must obey His rule wholeheartedly,	Declarative	Indirect directive	presenting the obligation of someone to do something	Assertion	Supporting information
Clause 07	and He said, “And fight them until there is no fitnah and the din is for Allah. But if they cease, then there is no aggression except against the tyrants” (Al-Baqarah 193).	Declarative	Statement of fact	telling about someone saying something	Logical-empirical	Supporting information
Clause 08	So the command of Allah– to which obedience is due as a religious duty – is to fight until there is no fitnah, i.e. evident shirk in the obedience of Allah, and until no manifest authority is given to any rule except to that of the True King.	Declarative	Indirect directive (necessity)	presenting the obligation of someone to do something	Duty	Main information
Clause 09	His saying, “if they cease,” means that if they submit or surrender, then they are to be left alone.	Declarative	Statement of opinion	presenting the meaning/the characteristic of something	Nature of situation	Supporting information
Clause 10	This can either be in the form of repentance to Allah and abiding by His worship alone or by the paying of jizyah while remaining upon subjugated kufr, just as Allah said, regarding the command of killing the mushrikin in general, “Then if they repent, establish the prayer, and pay the zakah, then leave them alone” (At-Tawbah 5), and He said that the People of the Book – those who “do not adhere to the true din” – should specifically be fought “until they pay the jizyah willingly, while they are humiliated” (At-Tawbah 29).	Declarative	Statement of opinion	presenting the meaning/the characteristic of something	Nature of situation	Supporting information
Clause 11	And “there is no aggression except against the tyrants” means those who did not submit nor surrender, either through repentance or dhimmah, the covenant of jizyah.	Declarative	Statement of opinion	giving an evaluative opinion of something	Nature of situation	Supporting information
Clause 12	Thus, anyone who is neither a Muslim nor a dhimmi kafir (while still a tyrant against himself, deserving both hatred and humiliation) is a hostile tyrant deserving aggression.	Declarative	Statement of opinion	giving an evaluative opinion of something	Nature of situation	Main information
Clause 13	This is because shirk itself is dhulm (tyranny), as Allah said, “And when Luqman said to his son, admonishing him, ‘O my son, do not commit shirk with Allah. Verily shirk is a great tyranny’” (Luqman 13).	Declarative	Statement of opinion	giving an evaluative opinion of something	Nature of situation	Supporting information
Clause 14	And although the people of dhimmah are mushrikin from the People of the Book, their shirk is subjugated and humiliated under the Shari'ah of Allah enforced upon them.	Declarative	Statement of opinion	giving an evaluative opinion of something	Nature of situation	Supporting information
Clause 15	So the duty to fight the tyrants – the mushrikin – is clear and established.	Declarative	Statement of opinion	giving an evaluative opinion of something	Duty	Main information
Clause 16	But Allah did not only command the “fighting” of disbelievers, as if to say He only wants us to conduct frontline operations against them.	Declarative	Statement of opinion	giving an evaluative opinion of something	Duty	Supporting information
Clause 17	Rather, He has also ordered that they be slain wherever they may be – on or off the battlefield.	Declarative	Statement of opinion	giving an evaluative opinion of something	Duty	Supporting information
Clause 18	He said, “So when the sacred months have passed, then kill the mushrikin wherever you find them and take them, surround them, and wait for them at every outpost” (At-Tawbah 5).	Declarative	Statement of fact	telling about someone saying something	Logical-empirical	Supporting information
Clause 19	All of this becomes all the more apparent for those who have realized that the blood of a kafir is cheap, filthy, and permissible to shed.	Declarative	Statement of opinion	giving an evaluative opinion of something	Nature of situation/disclaimer	Supporting information
Clause 20	Islam is the religion of sound principles providing the perfect foundations upon which the solid structures of justice and glory are built.	Declarative	Statement of opinion	presenting the meaning/the characteristic of something	Nature of situation	Supporting information
Clause 21	One of these great principles is that all people must be fought until they accept Islam or come under a shar'i covenant.	Declarative	Indirect directive	presenting the obligation of someone to do something	Nature of situation/duty	Main information
Clause 22	This principle establishes the prohibition of shedding Muslim and covenant-bound kafir blood as well as the permissibility of shedding the blood of all other kuffar.	Declarative	Indirect directive	Presenting prohibition of doing something	Nature of situation	Supporting information
Clause 23	The Prophet said, “I have been ordered to fight mankind until they say that there is no god except Allah and that I am the Messenger of Allah, and they establish the prayer and pay the zakah. Whoever does so, then his blood and wealth are safe from me except for a law- ful reason” (Reported by al-Bukhari and Muslim from Ibn ‘Umar), and he said, addressing the Muslims, “For verily your blood, wealth, and honor are *haram* to each other” (Reported by al-Bukhari and Muslim from Abu Bakrah).	Declarative	Statement of fact	telling about someone saying something	Logical-empirical	Supporting information
Clause 24	And regarding the dhimmi – the one who has a covenant with the Muslims –the Prophet said, “Whoever kills a person of covenant shall not smell the fragrance of Jannah, which can be found for a distance of forty years” (Reported by al-Bukhari and Muslim from ‘Abdullah Ibn ‘Amr).	Declarative	Statement of fact	telling about someone saying something	Logical empirical/duty	Main information
Clause 25	These narrations offer an explanation of the ayah, “And do not kill a soul which Allah has forbidden except for a lawful reason” (Al-An'am 151), about which at-Tabari said, “The soul which Allah has forbidden is the soul of a believer or a person of covenant, and His saying, ‘except for a lawful reason,’ means that which permits such a soul to be killed, as in capital punishment for murder, or stoning an adulterer to death, or killing someone for apostasy.”	Declarative	Statement of opinion	giving an evaluative opinion of something	Duty	Supporting information
Clause 26	As for all others – meaning all disbelievers who have no covenant – then their blood has not been given the sanctity of prohibition, but remains under the command to fight mankind, so their blood remains halal.	Declarative	Statement of opinion	giving an evaluative opinion of something	Nature of situation/duty	Main information
Clause 27	Shedding the blood of a non-dhimmi kafir is not sinful, but is rather rewarded with Jannah.	Declarative	Statement of opinion	giving an evaluative opinion of something	Nature of situation/expertise (positive)	Supporting information
Clause 28	Allah's Messenger said, “A kafir and his killer will never be gathered together in the Fire” (Reported by Muslim from Abu Hurayrah).	Declarative	Statement of fact	telling about someone saying something	Logical-empirical	Supporting information
Clause 29	Furthermore, his words, “I have been ordered to fight mankind,” leave no room for debate, as mankind includes every person in the world,	Declarative	Statement of opinion	giving an evaluative opinion of something	Nature of situation	Supporting information
Clause 30	and the only ones excluded from this order to fight are those who submit or surrender to the rule of Islam, as was previously mentioned.	Declarative	Statement of opinion	giving an evaluative opinion of something	Nature of situation	Supporting information
Clause 31	This alone befits a messenger who was sent to all people, as Allah β said, “Say, ‘O mankind, verily I am the Messenger of Allah sent to all of you’” (Al-A'raf 158),	Declarative	Statement of opinion	giving an evaluative opinion of something	Nature of situation	Supporting information
Clause 32	and the Prophet said, “Other prophets were sent to their people alone, but I was sent to all of mankind” (Reported by al-Bukhari from Jabir Ibn ‘Abdillah).	Declarative	Statement of fact	telling about someone saying something	Logical-empirical	Supporting information
Clause 33	Lest someone think this is a strange, new opinion, it should be known that this is the stance of the Sahabah and the greatest scholars of the Ummah.	Declarative	Indirect request	Giving suggestion	Suggestion.nature of situation	Main information
Clause 34	This is reflected in the words of ‘Umar Ibn al-Khattab who, during the life of the Prophet and without objection, said to Abu Jandal, encouraging him to kill his father Suhayl Ibn ‘Amr, “Be patient, Abu Jandal, for they are merely mushrikin, and their blood is nothing but (like) the blood of a dog” (Reported by Ahmad from al-Miswar Ibn Makhramah).	Declarative	Statement of opinion	giving an evaluative opinion of something	Logical empirical/duty	Supporting information
Clause 35	Indeed, ‘Umar was correct, as the mushrikin are entirely impure, as Allah said, “O you who believe! The mushrikin are nothing but impure” (At-Tawbah 28).	Declarative	Statement of opinion	giving an evaluative opinion of something	Nature of situation	Supporting information
Clause 36	This is also understood from the words of Anas Ibn Malik, who was asked, “O Abu Hamzah! What makes a slave's (i.e. a person's) blood and wealth *haram*?”	Declarative	Statement of opinion	giving an evaluative opinion of something	Nature of situation	Supporting information
Clause 37	He replied, “Whoever testifies that there is no god but Allah, faces our qiblah, prays our prayer, and eats what we slaughter, then he is a Muslim. He gets what a Muslim gets and is bound by what a Muslim is bound” (Reported by al-Bukhari from Maymum Ibn Siyah).	Declarative	Statement of fact	telling about someone saying something	Logical-empirical	Supporting information
Clause 38	Ash-Shafi'i said, “And the kafir's blood is not spared until he becomes a Muslim” (Al-Umm).	Declarative	Statement of fact	telling about someone saying something	Logical-empirical	Supporting information
Clause 39	In further explanation, he said, “Allah spared blood and restricted the taking of wealth, except for a lawful reason, through belief in Allah and in His Messenger or through a covenant given by the believers, based on the rule of Allah and His Messenger, to the People of the Book.	Declarative	Statement of fact	telling about someone saying something	Logical-empirical	Supporting information
Clause 40	And He allowed shedding the blood of mature men who refrain from belief and who do not have a covenant.	Declarative	Statement of opinion	giving an evaluative opinion of something	Duty	Supporting information
Clause 41	Allah said, ‘So when the sacred months have passed, then kill the mushrikin wherever you find them and take them, surround them, and wait for them at every out- post’ (At-Tawbah 5)” (Al-Umm).	Declarative	Statement of fact	telling about someone saying something	Logical-empirical	Supporting information
Clause 42	Mentioning the exception of killing women and children, he also said, “And the reason for the prohibition of shedding Muslim blood being different from the prohibition of shedding the kafir child's and kafir woman's blood is that they are not to be killed due to specific revelation restricting this killing [while the initial ruling allows shedding kafir blood in general].	Declarative	Statement of fact	telling about someone saying something	Logical-empirical	Supporting information
Clause 43	And our opinion regarding this – and Allah knows best – is that the restriction exists so that they can become slaves, which is more beneficial than killing them, and killing them does not harm the enemy; so making them slaves is more optimal than killing them” (Al-Umm).	Declarative	Statement of opinion	giving an evaluative opinion of something	Nature of situation	Main information
Clause 44	Al-Khattabi said, “The kafir's blood is permissible because he has not yet uttered the word of tawhid; but if he were to say it, then his blood is spared and becomes prohibited” (A'lam al-Hadith).	Declarative	Statement of fact	telling about someone saying something	Logical-empirical	Supporting information
Clause 45	Ibn Hazm, after mentioning the prohibition of targeting women and children, said, “And it is permissible to kill anyone – other than those we mentioned above – of the mushrikin, both the combatant and non-combatant, the businessman and employee, the elderly man – whether or not he be a person of influence – the farmer, the bishop or priest or monk, the blind or crippled – no one is given exception” (Al-Muhalla).	Declarative	Statement of fact	telling about someone saying something	Logical-empirical	Supporting information
Clause 46	Ibn Qudamah mentioned the harbi (the kafir who is not under a covenant) and said, “Shedding his blood is permissible without exception, just like swine” (Al-Mughni).	Declarative	Statement of fact	telling about someone saying something	Logical-empirical	Supporting information
Clause 47	He also said, “The kuffar asliyyin [those kuffar who are not murtaddin] shall have no protection in their own lands” (Al-Mughni).	Declarative	Statement of fact	telling about someone saying something	Logical-empirical	Supporting information
Clause 48	Ibn Rushd said, “And the principle is that what makes wealth permissible to take is kufr, and that which protects it is Islam, just as the Prophet γ said, ‘Then if they said that, they have protected their blood and their wealth from me’” (Bidayat al-Mujtahid).	Declarative	Statement of fact	telling about someone saying something	Logical-empirical	Supporting information
Clause 49	While specifically mentioning wealth, his words clearly point to the permissibility of bloodshed as is also in the evidence he quoted.	Declarative	Statement of opinion	giving an evaluative opinion of something	Nature of situation	Main information
Clause 50	Al-Qurtubi, said, “If a Muslim meets a kafir who has no covenant, it is permissible for him to kill him.”	Declarative	Statement of fact	telling about someone saying something	Logical-empirical	Supporting information
Clause 51	Abu Hanifah said, regarding a kafir who is killed, “There is no retaliation (against the killer) and the diyah (blood money) is not to be paid, because the blood of the kafir is permissible (to shed) unless it is established that he has a covenant or is under dhimmah” (Al-Hawi al-Kabir).	Declarative	Statement of fact	telling about someone saying something	Logical-empirical	Supporting information
Clause 52	Like-wise, the famous Hanafi scholar al-Kashani said, “The principle is that it is permissible to kill anyone from amongst the people who are at war (with the Muslims), whether they fight or do not fight.	Declarative	Statement of fact	telling about someone saying something	Logical-empirical	Supporting information
Clause 53	But it is not permissible to kill anyone who is not from the people who are at war [meaning those with a valid covenant], unless they fight or offer strategic support, obedience, instigation, or otherwise.	Declarative	Indirect directive	Presenting prohibition of doing something	Assertion	Main information
Clause 54	So the priest and wandering ascetic who mix with the people are to be killed, as are those who have fits of insanity, and the deaf and mute, and the one with a severed hand or foot, even if they did not fight.	Declarative	Indirect directive	presenting the obligation of someone to do something	Assertion	Supporting information
Clause 55	This is because they are from the same people who are at war [with the Muslims]” (Badai’ as-Sanai’).	Declarative	Statement of opinion	giving an evaluative opinion of something	Nature of situation	Supporting information
Clause 56	None of this should be surprising to any Muslim who has studied his religion, as this matter of a kafir's blood being halal to shed is something upon which scholars have recorded consensus.	Declarative	Statement of opinion	giving an evaluative opinion of something	Nature of situation	Main information
Clause 57	At-Tabari said, “They [the scholars of Islam] have agreed that even if a mushrik were to wear the bark of all the trees of the Haram [in Makkah] around his neck and on his arms, that would not grant him safety from being killed unless the Muslims gave him a covenant of dhimmah or security.”	Declarative	Statement of fact	telling about someone saying something	Logical-empirical	Supporting information
Clause 58	Muslims currently living in Dar al-Kufr must be reminded that the blood of the disbelievers is halal, and killing them is a form of worship to Allah, the Lord, King, and God of mankind.	Declarative	Indirect directive	presenting the obligation of someone to do something	Assertion	Main information
Clause 59	This includes the businessman riding to work in a taxicab, the young adults (post-pubescent “children”) engaged in sports activities in the park, and the old man waiting in line to buy a sandwich.	Declarative	Statement of opinion	giving an evaluative opinion of something	Nature of situation	Supporting information
Clause 60	Indeed, even the blood of the kafir street vendor selling flowers to those passing by is halal to shed	Declarative	Statement of opinion	giving an evaluative opinion of something	Nature of situation/assertion	Supporting information
Clause 61	– and striking terror into the hearts of all disbelievers is a Muslim's duty.	Declarative	Statement of opinion	giving an evaluative opinion of something	Assertion/Duty	Supporting information
Clause 62	There is no shar'i requirement to target soldiers and policemen nor judges and politicians,	Declarative	Statement of opinion	giving an evaluative opinion of something	Nature of situation	Supporting information
Clause 63	but all kuffar who are not under the covenant of dhimmah are fair game.	Declarative	Statement of opinion	giving an evaluative opinion of something	Nature of situation/assertion	Supporting information
Clause 64	How can the disbelievers ever dream of safety and security while Muslims suffer anywhere in the world and while the rule of Allah is mockingly replaced by manmade monstrosities of democracy?	Interrogative	Indirect directive	asking “How can someone do something, while something is in certain situation?”	Moral appeal	Main information
